# Supplementary material for: Multimodal Imaging Brain Connectivity Analysis (MIBCA) toolbox
Source: PeerJ. 2015 Jul 14;3:e1078. doi: 10.7717/peerj.1078 (PMC4511822; doi:10.7717/peerj.1078)
Supplement: Supplemental Information 1 — Blue fibers—Direct structural/functional connections; Red fibers—Mediated functional connections. [file peerj-03-1078-s001.pdf]

Annex I

Figure 1: Multimodal Connectogram (Interactive pdf). Blue fibers - Direct structural/functional connections; Red fibers - Mediated functional connections.
